# Supplementary material for: Association of endothelial dysfunction with sarcopenia and muscle function in a relatively young cohort of kidney transplant recipients
Source: PeerJ. 2021 Nov 22;9:e12521. doi: 10.7717/peerj.12521 (PMC8614188; doi:10.7717/peerj.12521)
Supplement: Supplemental Information 2 [file peerj-09-12521-s002.docx]

| **Code Name** | **Variable** |
| --- | --- |
| Number | Participant number |
| Age | Age (years) |
| Sex | Sex (1=male; 2=female) |
| KT_duration | Time from transplantation (months) |
| Smoking | Current smoking (0=No; 1=Yes) |
| SBP | Systolic blood pressure (mmHg) |
| DBP | Diastolic blood pressure (mmHg) |
| Vascular_reactive_index | Vascular reactivity index |
| VRI_group | VRI group (1=good, VRI ≥ 2.0; 2=intermediate, 1.0 ≤ VRI < 2.0; 3=poor, VRI < 1.0) |
| BMI | Body mass index (kg/m^2^) |
| Body_fat | Body fat (%) |
| Muscle_mass | Skeletal muscle mass (kg) |
| SMI | skeletal muscle index (kg/m^2^) |
| Low_SMI | Low SMI group (0=normal; 1=low) |
| HGS | Handgrip strength (kg) |
| HGS_groups | HGS group (0=normal; 1=low) |
| Gait_speed | Gait speed (ms) |
| Gait_speed_group | Gait speed group (0=normal; 1=low) |
| Sarcopenia | Sarcopenia (0=No; 1=Yes) |
| Hb | Hemoglobin (g/dL) |
| TCH | Total cholesterol (mg/dL) |
| TG | Triglyceride (mg/dL) |
| GluAC | Fasting glucose (mg/dL) |
| IP | Creatinine (mg/dL) |
| Cre | Phosphorus (mg/dL) |
| iPTH | Intact PTH (pg/mL) |
| DM | Diabetes mellitus (0=No; 1=Yes) |
| Hypertension | Hypertension (0=No; 1=Yes) |
| Hyperlipidemia | Hyperlipidemia (0=No; 1=Yes) |
| CV_disease | Cardiovascular disease (0=No; 1=Yes) |
| Tacrolimus | Tacrolimus (0=user; 1=non-user) |
| Mycophenolic acid | Mycophenolic acid (0=user; 1=non-user) |
| Steroids | Steroids (0=user; 1=non-user) |
| Rapamycin | Rapamycin (0=user; 1=non-user) |
| Cyclosporin | Cyclosporin (0=user; 1=non-user) |
